# Strategies for assessing and preventing cardiovascular disease risk in inflammatory bowel disease patients: A meta-analysis and meta-regression and bibliometric review

**DOI:** 10.1371/journal.pone.0327734

**Published:** 2025-07-28

**Authors:** Lu-Fei Liu, Yi-Wei Fan, Ye Lv, Zhao-Xia Liu, Xiao-Ce Dai

**Affiliations:** 1 Heilongjiang University of Chinese Medicine, Harbin City, Heilongjiang Province, China; 2 Department of Emergency, Zhuhai People’s Hospital, Zhuhai Hospital Affiliated with Jinan University, The First Affiliated Hospital of Faculty of Medicine Macau University of Science & Technology, Zhuhai, Guangdong, China; 3 Department of Gynecology, Zhuhai People’s Hospital, Zhuhai Hospital Affiliated with Jinan University, The First Affiliated Hospital of Faculty of Medicine Macau University of Science & Technology, Zhuhai, Guangdong, China; 4 Department 1 of Digestion, First Affiliated Hospital, Heilongjiang University of Chinese Medicine, Harbin City, Heilongjiang Province, China; 5 Department of Cardiology, Zhejiang Hospital, School of Medicine, Zhejiang University, Hangzhou, Zhejiang, China; Noorda College of Osteopathic Medicine, UNITED STATES OF AMERICA

## Abstract

**Objective:**

To evaluate how inflammatory bowel disease in adults increases the risk of developing cardiovascular disease.

**Design:**

Meta-analysis, meta-regression and bibliometric review.

**Data sources:**

PubMed and Embase were searched from their inception until December 19, 2024. Additionally, a manual search was conducted to identify cohort studies and related systematic reviews. Web of Science Core Collection (WoSCC) database was used to bibliometric analysis.

**Eligibility criteria:**

Cohort studies assessing the risk of cardiovascular disease (CVD) in adults with inflammatory bowel disease (IBD) were included. Non inflammatory bowel disease exposure was considered as the control group. The risk of bias and the certainty of the evidence were evaluated. Meta analysis, meta regression, sensitivity analysis, and bibliometric analysis of keywords were performed.

**Results:**

23 studies, comprising 20 different samples (n=45,887,104), were included. meta-analyses showed adults with IBD regardless of Crohn’s disease and ulcerative colitis increased the CVD risk. This risk increased in IBD patients after conducting sensitivity analyses. However, the strength of evidence was deemed very low for all outcomes with substantial heterogeneity observed across all outcomes. The bibliometric review showed the neutrophil-to-lymphocyte ratio can act as a biomarker for predicting IBD severity.

**Conclusion:**

While inflammatory bowel disease (IBD) may elevate the risk of cardiovascular disease (CVD), current evidence does not support the use of medication for primary prevention. Randomized controlled trials are needed to address this gap in knowledge.

## Introduction

Cardiovascular disease (CVD) is the leading cause of mortality and morbidity, resulting in more premature deaths than cancer [1–3]. To reduce the incidence of CVD, it is crucial to enhance prevention efforts targeted at young adults [4]. While standard modifiable cardiovascular risk factors—such as hypertension, diabetes mellitus, hyperlipemia, and tobacco—are known contributors to the development of CVD, recent studies have shown that some individuals who experience acute myocardial infarction (AMI) do not exhibit these factors [5]. Individuals suffering from conditions like human immunodeficiency virus (HIV) infection, psoriasis, and rheumatoid arthritis face a heightened likelihood of experiencing accelerated atherosclerosis [6–8]. Chronic inflammation has been identified as a significant contributor to the development of CVD [9]. Recent clinical trials including CANTOS (Canakinumab Anti-Inflammatory Thrombosis Outcome Study) and COLCOT (Colchicine Cardiovascular Outcomes Trial) have provided evidence of the causal relationship between systemic inflammation and ASCVD [10,11]. Therefore, identifying new cardiovascular risk factors may be essential for effective disease prevention. Certain typical conditions and characteristics of working-age adults, including chronic inflammation and adverse gestational factors, are independently associated with an increased risk of developing CVD [8,12,13]. These factors have gained recognition in recent years as promising candidates for personalized and timely preventive strategies.

Research has shown that females over the age of 40 with IBD, have a higher risk of myocardial infarction (MI) compared to males [14,15]. This finding suggests that the non-menopausal period may not serve as a protective factor against the development of cardiovascular disease (CVD) in women with IBD. Interestingly, tobacco, a well-known risk factor for CVD, plays different roles in Crohn’s disease (CD) and ulcerative colitis (UC): it accelerates the development and progression of CD and leads to a poor response to both medical and surgical treatments, while exhibiting protective effects in UC.

The 2018 guideline on blood cholesterol management indicate that fasting lipid profiles can serve as indicators for evaluating the effectiveness of statin therapy in patients with chronic inflammatory disorders undergoing disease-modifying therapy or antiretroviral therapy [16]. In the following year, the ESC guidelines for the management of dyslipidemias clarified that the use of lipid-lowering drugs should not be based solely on the presence of chronic immune-mediated inflammatory diseases [17]. However, a significant challenge persists: there are currently no established indicators that show statin therapy actually benefits these patients.

Previous meta-analyses have indicated that IBD may elevate the risk of CVD; [18–20] however, several issues have been identified: 1) no meta-analysis has explained the substantial heterogeneity among studies, which can approach 100% I²; and 2) no meta-analysis has evaluated the strength of the evidence linking IBD to CVD outcomes. Furthermore, for IBD patients without other cardiovascular risk factors, clinical evidence for whether statins should be used for primary prevention still requires further investigation.

We conducted a reevaluation of the meta-analysis to examine the relationship between IBD and CVD risk through meta-regression, while excluding outliers to reduce heterogeneity and provide a prediction interval for the effects of future studies. Additionally, we utilized a bibliometric approach to identify recent potential testing indices for assessing the severity of IBD.

## Method

This meta-analysis was conducted to the Preferred Reporting Items for Systematic Reviews and Meta-Analyses (PRISMA) guidelines (S1 File) [21]. The protocol was registered at PROSPERO and its number was CRD42025630206.

### Search strategy

A systematic search of PubMed and Embase to identify published studies was performed from inception to December 19, 2024. Electronic searches were used by exploded Medical Subject Headings (MeSH) terms and related key words. The search terms used were (MeSH exp “Inflammatory Bowel Diseases” and key words “Inflammatory Bowel Disease*”) and (MeSH exp “Colitis, Ulcerative” and key words “Colitis, Ulcerative” and “Ulcerative Colitis” and “Colitis Gravis”) and (MeSH exp “Crohn Disease” and key words “Crohn Disease*” and “Crohn’s Enteritis” and “Regional Enteritis” and “Crohn’s Disease” and “Crohns Disease” and “Enteritis, Granulomatous” and “Granulomatous Enteritis” and “Ileocolitis” and “Granulomatous Colitis” and “Regional Ileitis”) and (MeSH exp “Cardiovascular Disease” and key words “Myocardial Infarction*” and “Cardiovascular Stroke” and “Myocardial Infarct*” and “NSTEMI” and “STEMI”). We also manually reviewed the reference lists of all relevant reviews to identify additional potential studies. The detailed search strategy for each of the databases was provided in PROSPERO protocol. We did not make language restrictions.

### Study selection

Two authors (X.-C. D. and Z.-X. L.) independently conducted the original search, struck out duplicate articles, read the relevant titles and abstracts and classified records as included, excluded or uncertain. Due to ambiguity, the full-text article was acquired to identify eligibility. If multiple studies used data from the same cohort or database, the most recent and most comprehensive article was included. Discussion and consensus were used to resolve discrepancies.

### Definition and PICOS of this study

We defined cardiovascular diseases to include coronary artery disease and stroke. The working-age population was categorized as individuals aged 18–75 years. Inflammatory bowel diseases were identified as comprising Crohn’s disease and ulcerative colitis.

P: IBD patients.
I/E: admissions for the reason of the first-time diagnosis of CVDs.
C: General populations.
O: total CVD events including coronary artery disease, hemorrhagic and ischemic stroke.
S: cohort studies.

### Data extraction

Data extraction was performed by X.-C. D. and checked separately by other authors (Y.-W. F. and Y. L.). Gathered data including the following: first author, year of publication, cohort name, study type, data source, participants, interventions, sex ratio, mean age, study period, IBD types, outcomes, outcome definition and adjusted factors. If unadjusted or adjusted RR or HR and 95% CIs were available, these data were collected. Co-authors resolved discrepancies by discussion.

### Risk of bias assessment

Two author (L.-F. L. and Y.-W. F.) independently assessed risk of bias using the Newcastle Ottawa Scale (NOS) for Quality Assessment for cohort and case-control studies [22]. NOS rates case-control studies on case definition, representativeness of cases, selection and definition of controls, comparability of controls, Ascertainment of exposure, same method for ascertainment of cases and controls and non-response rate. NOS rates cohort studies on representativeness of the exposed cohort, representativeness of the non-exposed cohort, ascertainment of exposure, outcome of interest was not present at the beginning of study, comparability of cohorts because of the design or analysis, assessment of outcome, follow-up long enough for outcomes to occur and adequacy of follow up of cohorts. Studies were considered as low quality (below 5 stars), moderate quality (5–7 stars’) and high quality (above 7 stars’).

### Certainty of the evidence

Two independent reviewers (Z.-X. L. and X.-C. D.) utilized the Grading of Recommendations, Assessment, Development, and Evaluations (GRADE) approach to assess the certainty of the evidence across five domains: risk of bias, imprecision, inconsistency, indirectness, and publication bias [23].

### Bibliometric analysis

A preliminary search was performed in the Web of Science Core Collection (WoSCC) database to identify relevant keywords. The retrieval criteria were set as follows: [(TS = “Inflammatory Bowel Disease”) OR (TS = “Crohn’s Disease”) OR (TS = “Ulcerative Colitis”)] AND (TS = “cardiovascular disease”), focusing exclusively on original research articles and reviews. The timespan covered 23 years, from January 1, 2000, to December 31, 2023, and the language was restricted to English. Citespace, VOSviewer, and Bibliometrix were employed to visualize keyword clusters using various methods.

### Data analysis

Random effects model with inverse variance method was used to pool studies’ data which were described in the above section. Subgroup analysis stratified by different MI types and IBD were conducted. We considered hazard ratios (HRs) to be similar to relative risks (RRs) and calculated RRs along with 95% confidence intervals (CIs) for the overall effect estimate. If the study only reports the odds ratios (OR), we convert OR to RR using: RR=OR/((1-N_events_/N_control_) + (OR* N_events_/N_control_)). Given our anticipation of substantial heterogeneity between studies, we employed a random-effects model to pool the effect sizes. The restricted maximum likelihood estimator was used to calculate the heterogeneity variance, and Knapp-Hartung adjustments were applied to determine the confidence intervals around the pooled effect. Data was pooled with an inverse variance weighting method. The assessment of heterogeneity among studies was conducted using I^2^ statistics, and Tau^2^ was additionally incorporated into the forest plots. P < .05 was considered statistically meaning for total included analyses, except where otherwise specified. Sensitivity analyses were conducted to identify outliers or influential cases through various methods, including influence analysis, leave-one-out methods, the Baujat plot, and exploratory data analysis. If any study was identified as an outlier or influential case, it was excluded from the meta-analysis. A prediction interval was included in the forest plots when the meta-analysis comprised at least three studies, accounting for the heterogeneity among the studies to evaluate the likelihood of observing true treatment effects in future settings [24]. Publication bias and the potential presence of small-study effects were assessed using a funnel plot, contour-enhanced funnel plot, Egger’s test (number of studies >10) and P curve. Meta-regressions were performed to examine potential sources of heterogeneity, considering covariates at the study level [25]. The following predictors were included: age, NOS, publication year, and sample size. All statistical analyses were conducted on R studio software (v. 4.1.1). Our findings are based solely on secondary analysis of published research data and do not involve ethical approval.

## Results

### Trial selection

**Fig 1:**
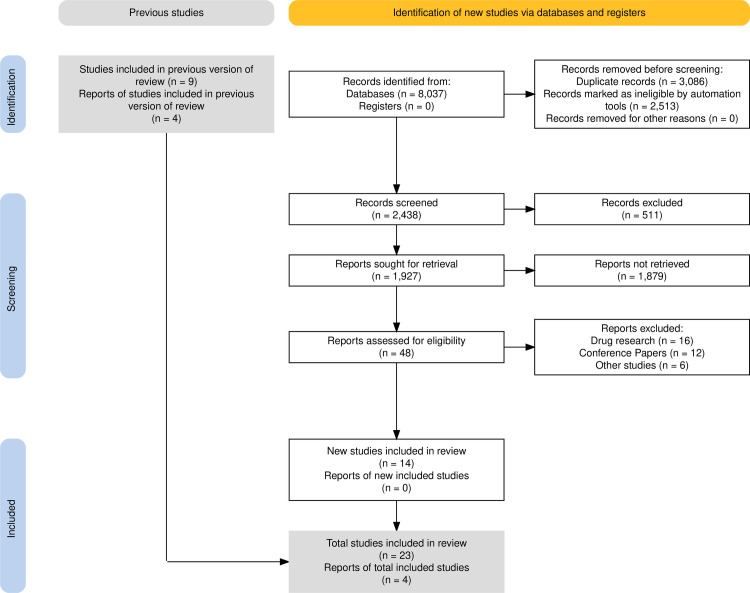
The PRISMA flowchart of this meta-analysis.

The total literature screening and study selection procedure is described in the PRISMA flowchart (Fig 1). The initial search yielded 8037 records from PubMed and EMBASE. Of these citations, forty-eight studies were thought to fulfill the inclusion criteria. After reviewing and assessing the full-text, 23 observational studies were eligible for this meta-analysis [15, 26–47].

### Description of included studies

The total number of participants was 45,887,104, with follow-up durations ranging from approximately 4–30 years. Most participants were of working age (18–75 years). Detailed characteristics of the included studies can be found in S1 Table.

### Risk of bias assessment

No studies were rated below 6 points (S2 Table), indicating moderate to high quality. Bias arose from insufficient control of important factors and inadequate sample size, particularly when studies did not clearly report their study design or adjust for confounding variables.

### Certainty of the evidence (GRADE)

Significant concerns were primarily identified regarding the risk of bias, indirectness of the evidence, inconsistency, and imprecision of the results. As a result, the certainty of the evidence for all outcomes was assessed as very low (S3 Table).

### Total inflammatory bowel disease and the risk for cardiovascular disease (GRADE: Very low evidence)

**Fig 2:**
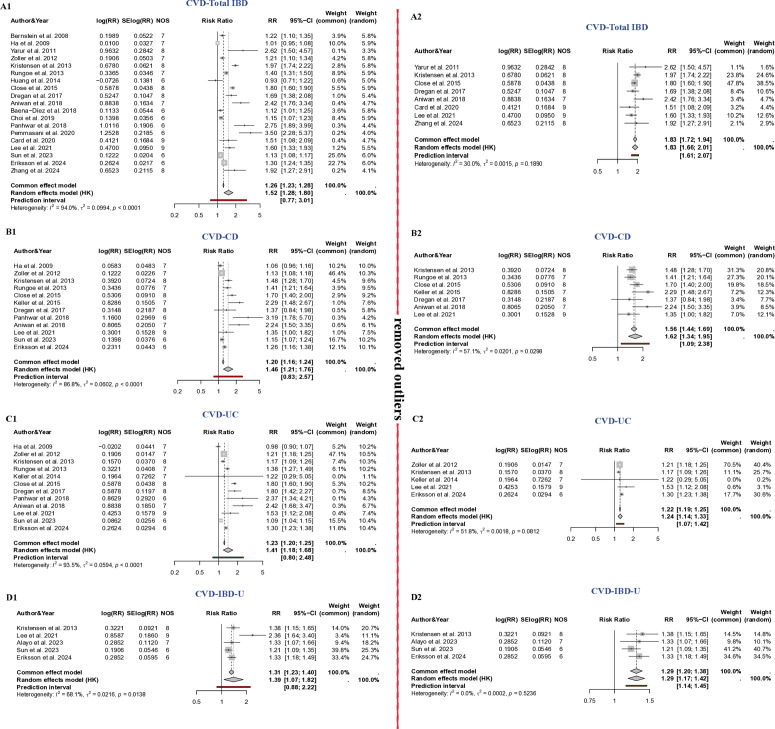
Forrest plot of inflammatory bowel disease and the increased cardiovascular disease risk. A1: the association between the total IBD and the increased CVD risk. A2: the association between the total IBD and the increased CVD risk after removing the outliers. B1: the association between CD and the increased CVD risk. B2: the association between CD and the increased CVD risk after removing the outliers. C1: the association between UC and the increased CVD risk. C2: the association between UC and the increased CVD risk after removing the outliers. D1: the association between IBD-U and the increased CVD risk. D2: the association between IBD-U and the increased CVD risk after removing the outliers.

A meta-analysis showed that total inflammatory bowel disease (IBD) increased the risk for cardiovascular disease (CVD), with a relative risk (RR) of 1.52 (95% CI: 1.28 to 1.80); I^2 ^= 94%; Tau^2^ = 0.1. Nineteen studies were included in this meta-analysis, evaluating the relationship between IBD and the risk of first-time diagnosis of CVD.(Fig 2A1) The outcome assessment primarily focused on the working-age IBD population, with 84% of the studies addressing this demographic.

Meta-regression analysis (S2 File) and sensitivity analyses (S1–S6 Figs) were conducted to identify sources of heterogeneity. In the meta-regression analysis, the Newcastle-Ottawa Scale (NOS) accounted for a significant portion of the heterogeneity (76.7%). Moreover, the combined effect of NOS, age, sample size, and publication year fully explained the observed heterogeneity. The between-study heterogeneity variance was estimated at Tau^2^ = 0.1 (95%CI: 0.05–0.27), with an I^2^ value of 94% (95%CI: 92–95.6%). The prediction interval ranged from RR = 0.77 to 3.01, indicating that negative association cannot be ruled out for future studies.

The sensitivity analysis, which included Baujat plots, influence plots, and the leave-one-out method, demonstrated that the substantial heterogeneity was not attributable to any single study (S1A, S2A and S3A Figs). Heterogeneity decreased after excluding 11 outliers with extreme effect sizes to mitigate their influence on the pooled studies. Following this exclusion, the relationship between IBD and the risk for CVD improved to a RR of 1.83 (95% CI: 1.66 to 2.01); I^2^ = 30%; Tau^2^ = 0.002. The prediction interval also adjusted from 1.61 to 2.07 after the removal of the outliers. (Fig 2A2)

Publication bias was detected, as indicated by Egger’s test (p = 0.02; S4-1 Table). The funnel plot (S4A Fig) appeared asymmetrical, and the contour-enhanced funnel plots (S5A Fig) indicated that only one study exhibited a small-study effect with statistical significance. To address small-study effects, a limited meta-analysis provided an adjusted estimate of RR 1.36 (95% CI: 1.15 to 1.61; p = 0.0003). Additionally, the P-curve (S6A Fig) indicated that the presence of publication bias was not due to p-hacking within the studies.

### Crohn’s disease and the risk for cardiovascular disease (GRADE: Very low evidence)

A meta-analysis showed that Crohn’s disease (CD) increased the risk for cardiovascular disease (CVD), with a relative risk (RR) of 1.46 (95% CI: 1.21 to 1.76); I^2 ^= 86.8%; Tau^2^ = 0.06. Twelve studies were included in this meta-analysis, evaluating the relationship between CD and the risk of first-time diagnosis of CVD.(Fig 2B1) The outcome assessment mainly focused on the working-age CD population, with 92% of the studies addressing this demographic.

In the meta-regression analysis (S1 File), sample size was found to explain the total heterogeneity, which was equal to the combined moderators of age, Newcastle-Ottawa Scale (NOS), sample size, and publication year.

The between-study heterogeneity variance was estimated at τ^2^ = 0.06 (95%CI: 0.03–0.28), with an I^2^ value of 86.8% (95%CI: 78.7–91.8%). The prediction interval ranged from *RR *= 0.83 to 2.57, indicating that negative association cannot be ruled out for future studies.

The series of sensitivity analysis demonstrated that the substantial heterogeneity was not attributable to any single study (S1B, S2B and S3B Figs). To reduce the extreme effect sizes among the pooled studies, heterogeneity decreased after excluding 4 outliers. Following this exclusion and omitting Eriksson et al., the relationship between CD and the risk for CVD increased to a RR of 1.62 (95% CI: 1.34 to 1.95); I^2^ = 57.1%; Tau^2^ = 0.02. The prediction interval also adjusted from 1.09 to 2.38 after the removal of the outliers. (Fig 2B2)

Publication bias was detected, as indicated by Egger’s test (p = 0.001; S4-2 Table). The funnel plot (S4B Fig) appeared asymmetrical, and the contour-enhanced funnel plots (S5B Fig) indicated that only one study exhibited a small-study effect with statistical significance. To address small-study effects, a limited meta-analysis provided an adjusted estimate of RR 1.23 (95% CI: 1.01 to 1.5; p = 0.0357). Additionally, the P-curve (S6B Fig) indicated that the presence of publication bias was not due to p-hacking within the studies.

### Ulcerative Colitis and the risk for cardiovascular disease (GRADE: Very low evidence)

A meta-analysis showed that Ulcerative Colitis (UC) increased the risk for cardiovascular disease (CVD), with a relative risk (RR) of 1.41 (95% CI: 1.18 to 1.68); I^2 ^= 93.5%; Tau^2^ = 0.06. Twelve studies were included in this meta-analysis, evaluating the relationship between UC and the risk of first-time diagnosis of CVD. (Fig 2C1)The outcome assessment mainly focused on the working-age UC population, with 92% of the studies addressing this demographic.

In the meta-regression analysis (S1 File), the Newcastle-Ottawa Scale (NOS) accounted for 44.71% of the heterogeneity. Additionally, the combined moderators of age, NOS, sample size, and publication year were able to fully explain the heterogeneity observed.

The between-study heterogeneity variance was estimated at Tau^2^ = 0.06 (95%CI: 0.02–0.22), with an I^2^ value of 93.5% (95%CI: 90.4–95.6%). The prediction interval ranged from RR = 0.8 to 2.48, indicating that negative association cannot be ruled out for future studies.

The sensitivity analysis revealed that the significant heterogeneity was not due to any single study (S1C, S2C and S3C Figs). To address the extreme effect sizes within the pooled studies, heterogeneity was reduced following the exclusion of seven outliers. After this exclusion, the association between UC and CVD risk decreased to a relative risk (RR) of 1.24 (95% CI: 1.14 to 1.33); I² = 51.8%; Tau² = 0.002. (Fig 2C2) Additionally, the prediction interval adjusted from 1.07 to 1.42 after the removal of the outliers.

Publication bias was not detected, as indicated by Egger’s test (p = 0.18; S4-3 Table). The funnel plot (S4C Fig) appeared symmetrical, and the contour-enhanced funnel plots (S5C Fig) showed no evidence of a small-study effect. Additionally, the P-curve (S6C Fig) suggested that there was no indication of p-hacking within the studies.

### Inflammatory bowel disease-unclassified and the risk for cardiovascular disease (GRADE: Very low evidence)

A meta-analysis showed that IBD-U increased the risk for CVD, with a relative risk (RR) of 1.39 (95% CI: 1.07 to 1.82); I^2 ^= 68.1%; Tau^2^ = 0.02. Five studies were included in this meta-analysis, evaluating the relationship between IBD-U and the risk of first-time diagnosis of CVD. (Fig 2D1) The outcome assessment mainly focused on the working-age IBD-U population, with 100% of the studies addressing this demographic. Only five studies were included, meta-regression analysis and publication bias could not be developed. The funnel plot (S4D Fig) appeared symmetrical.

The between-study heterogeneity variance was estimated at Tau^2^ = 0.02 (95%CI: 0.002–0.56), with an I^2^ value of 68.1% (95%CI: 17.6–87.6%). The prediction interval ranged from RR = 0.88 to 2.22, indicating that negative association cannot be ruled out for future studies.

The sensitivity analysis revealed that the significant heterogeneity is from Lee et al. (S1D–S3D Figs). After this exclusion, the association between IBD-U and CVD risk decreased to a relative risk (RR) of 1.29 (95% CI: 1.20 to 1.38); I² = 0; Tau² = 0.0002. Additionally, the prediction interval adjusted from 1.14 to 1.45 after the removal of the outliers (Fig 2D2). Additionally, the P-curve (S6D Fig) suggested that there was no indication of p-hacking within the studies.

### Inflammatory bowel disease and the risk for acute myocardial infarction (GRADE: Very low evidence)

**Fig 3:**
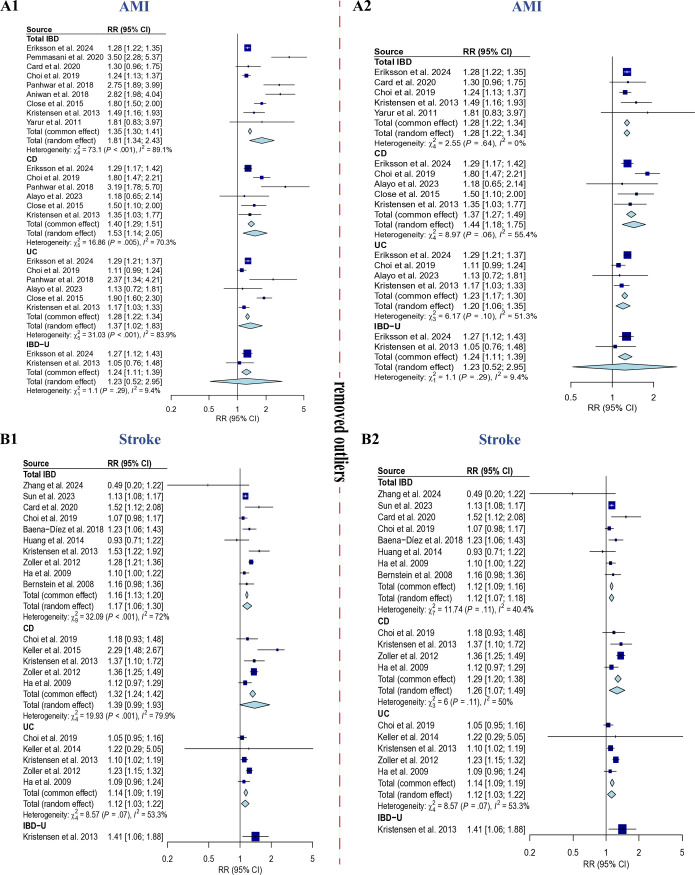
Forrest plot of inflammatory bowel disease and the increased myocardial infarction and stroke risk. A1: the association between the total IBD, CD, UC, and IBD-U and the increased MI risk. A2: the association between the total IBD, CD, UC, and IBD-U and the increased MI risk after removing the outliers. B1: the association between the total IBD, CD, UC, and IBD-U and the increased stroke risk. B2: the association between the total IBD, CD, UC, and IBD-U and the increased stroke risk after removing the outliers.

A meta-analysis indicated that inflammatory bowel disease (IBD) significantly increased the risk of acute myocardial infarction (AMI), with a relative risk (RR) of 1.81 (95% CI: 1.34 to 2.43); I² = 89.1%; P_heterogeneity_ < 0.001. Furthermore, Crohn’s disease (CD), ulcerative colitis (UC), and IBD-unclassified (IBD-U) also elevated the AMI risk, with RRs of 1.53 (95% CI: 1.14 to 2.05); I² = 70.3%; P_heterogeneity_ < 0.005, 1.37 (95% CI: 1.02 to 1.83); I² = 83.9%; P_heterogeneity_ < 0.001, and 1.24 (95% CI: 1.11 to 1.39); I² = 9.4%; P_heterogeneity_ = 0.29, respectively. (Fig 3A1) Due to the limited number of studies, meta-regression analysis and assessment of publication bias could not be performed. After removing outliers, all pooled relative risks decreased, suggesting that the substantial heterogeneity may have overestimated the pooled relative risks. Following adjustment, different types of IBD were associated with a 23% to 44% increase in AMI risk. (Fig 3A2)

### Inflammatory bowel disease and the risk for stroke (GRADE: Very low evidence)

A meta-analysis revealed that inflammatory bowel disease (IBD) significantly increased the risk of stroke, with a relative risk (RR) of 1.17 (95% CI: 1.06 to 1.30); I² = 72%; P_heterogeneity_ < 0.01. In contrast, Crohn’s disease (CD) showed no association with stroke risk, yielding an RR of 1.39 (95% CI: 0.99 to 1.93); I² = 79.9%; P_heterogeneity_ < 0.001. Additionally, ulcerative colitis (UC) and IBD-unclassified (IBD-U) were found to elevate stroke risk, with RRs of 1.14 (95% CI: 1.09 to 1.19); I² = 53.3%; P_heterogeneity_ = 0.07, and 1.41 (95% CI: 1.06 to 1.88), respectively. (Fig 3B1) Due to the limited number of studies, meta-regression analysis and assessment of publication bias could not be conducted. After removing outliers, all pooled relative risks decreased, suggesting that the substantial heterogeneity may have led to an underestimation of the pooled relative risks. Following adjustment, different types of IBD were associated with a 12% to 29% increase in stroke risk (excluding IBD-U). (Fig 3B2).

### Exploratory subgroup analysis

Exploratory subgroup analyses showed significant heterogeneity (almost 100%) between groups (S5 Table). According to continental subgroups, we found that patients with IBD in North America had a significantly higher risk of developing CVD than those in Asia and Europe (P for interaction = 0.0065). Additionally, the subgroups adjusted for risk factors were statistically different from the non-risk factor-adjusted subgroups (P for interaction < 0.001). There was no statistically significant difference in the remaining subgroups.

### Analysis of keywords

**Fig 4:**
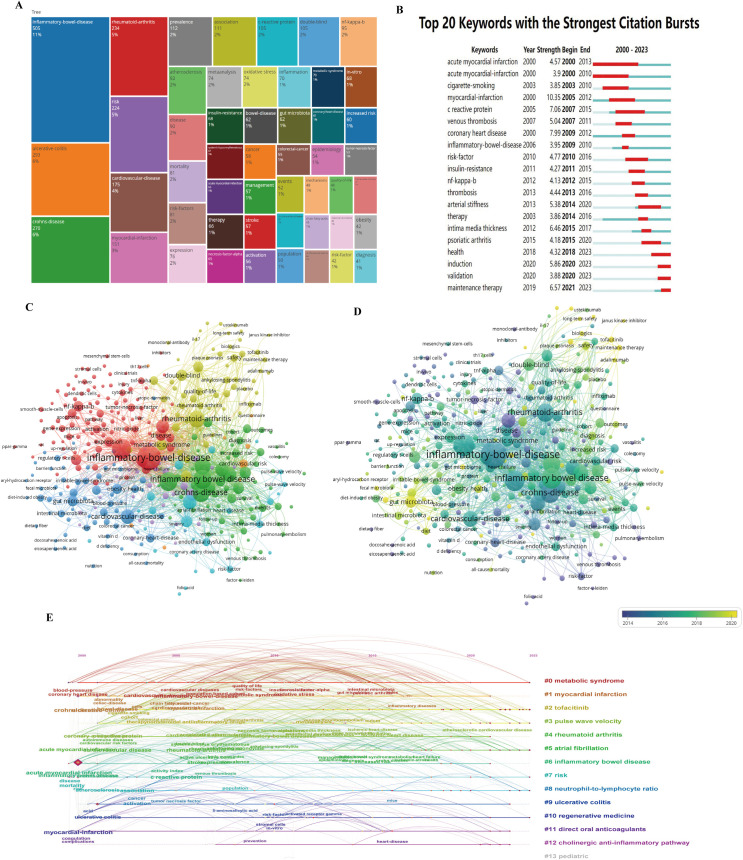
The keywords analysis. A. the tree map of the frequent used keywords. B. Top 20 keywords with strongest citation bursts. C. the cluster of frequent used keywords. D. the cluster of frequent used keywords with time effect. E. The time cluster of frequent used keywords.

Keywords effectively convey the main topic of an article, and analyzing these keywords can reveal research hotspots and trends within a specific field. After extracting and merging the keywords, the most frequent terms were displayed in a tree map (Fig 4A). Burst detection results are illustrated in Fig 4B, which lists the top twenty keywords with the strongest citation bursts. The keyword “acute myocardial infarction” exhibited the strongest citation burst in 2010. Other recent keywords with significant citation bursts include “health,” “induction,” “validation,” and “maintain therapy.”

The network visualization maps (Fig 4C and D) depict keyword clusters and their temporal effects, with earlier occurrences represented by lighter colors and later occurrences indicated by darker shades. The timeline graph (Fig 4E) organizes keywords chronologically to illustrate the evolution of research hotspots and development processes. Notable topics include #3 pulse wave velocity, #5 atrial fibrillation, #8 neutrophil-to-lymphocyte ratio, and #10 regenerative medicine.

## Discussion

This meta-analysis aimed to assess how inflammatory bowel disease (IBD) impacts the risk of developing cardiovascular disease (CVD). After analyzing a total of 23 studies (comprising 20 different samples), we found that patients with IBD had an increased risk of CVD during their working age. Patients with Crohn’s disease (CD) were found to have a higher risk of CVD, acute myocardial infarction, and stroke compared to patients with ulcerative colitis (UC) after conducting a series of sensitivity analyses, in which certain studies identified as outliers or sources of heterogeneity were excluded. We had mentioned that the previous meta-analyses had some limitations (**see** **Introduction**). The study design like cross-sectional study, and type of included population were the innate heterogeneity among studies. Although some studies demonstrated a positive association between IBD and AMI, they utilized inpatient databases; [48] therefore, the conclusion that IBD increased the risk of AMI cannot be definitively drawn. It was more precise to assert that patients with IBD requiring hospitalization are at a heightened risk of AMI compared to those who do not require inpatient treatment. This conclusion was substantiated by a study included in this meta-analysis, which examined 20,795 patients from Denmark and demonstrated that the risks of AMI, stroke, and cardiovascular mortality were significantly increased during periods of IBD flare, whereas the risk during remission was comparable to that observed in control subjects [42]. Furthermore, the risk of CVD appears to be significantly elevated during the first year following the diagnosis of inflammatory bowel disease (IBD), likely associated with disease activity. However, this increased risk does not seem to correlate with an increased rate of cardiovascular mortality [49,50]. Although this interesting phenomenon has been identified, the existing clinical evidence is not robust and necessitates further research to gain a more comprehensive understanding of this association.

Research has demonstrated that the activity of IBD can serve as an indicator for the accelerated development of cardiovascular disease CVD [42]. Therefore, it is essential to promote the optimization of IBD management in all patients, particularly during active flare-ups. It is worth mentioning that disease-modifying therapies, including anti-inflammatory and immunomodulatory agents, can play a role in reducing CVD risk in patients with IBD. A large cohort study revealed that the use of 5-aminosalicylic acid was associated with a reduced risk of ischemic heart disease [43]. Notably, this protective effect was found to be dose-dependent and was observed exclusively in patients taking corticosteroids, which acted as an indicator of disease severity. Additionally, another observational study indicated that the use of anti-TNF agents was associated with a reduced risk of acute arterial events [51]. These findings suggest that the CVD risk is particularly pronounced in the early years following IBD diagnosis. However, this risk appears to be mitigated when corticosteroids are administered within a certain dose range, or when other modifying therapies are consistently utilized.

The question about the relationship between the risk of inflammatory bowel disease (IBD) and cardiovascular disease (CVD) becomes straightforward: can increasing the use of statins or/and aspirin during IBD episodes effectively reduce this risk to a level comparable to the general population? Unfortunately, despite recent primary prevention guidelines indicating that chronic inflammatory diseases, including IBD, operate through independent mechanisms leading to CVD risk [17,52], these conditions should be viewed primarily as risk enhancers or modulators rather than independent diseases warranting specific primary prevention measures [17,52]. Therefore, the challenge still lies in addressing the unique CVD risk associated with IBD, rather than categorizing it as an independent disease that requires primary prevention strategies.

The main clinical finding from the meta-analysis and bibliometric review indicates that inflammatory bowel disease may be positively associated with an increased risk of cardiovascular disease. Additionally, the neutrophil-to-lymphocyte ratio has emerged as a valuable biomarker for predicting disease severity in IBD patients [53], and it could also be incorporated into cardiovascular disease risk assessments. Although numerous cohort studies have indicated that inflammatory bowel disease, as a type of chronic inflammatory disease, can trigger the development of cardiovascular diseases—particularly during acute stages like myocardial infarction and stroke—these studies often lacked a precise definition of the population studied [29,35,36]. This ambiguity may lead to an overestimation of the risk for cardiovascular disease, particularly as it may encompass a significant portion of individuals drawn from hospital settings. Moreover, no causal relationship between IBD and CVD has been established [54]. Our previous study suggested that gut microbiota dysfunction was causally associated with cardiovascular diseases [54]. This perspective supports the mechanism by which IBD may contribute to CVD, as IBD leads to immune dysfunction, resulting in microbiome abnormalities that increase the risk of thromboembolic events [55]. Currently, despite the interesting clinical findings, caution must be exercised before supporting statins or aspirin as the primary preventive measure for patients diagnosed with inflammatory bowel disease only. The overall certainty of the evidence was evaluated as very low, suggesting that the existing evidence does not warrant the recommendation of primary prevention strategies for patients with inflammatory bowel disease.

### Limitations

We implemented a rigorous search strategy and established specific inclusion criteria; consequently, some potentially relevant studies may have been overlooked or excluded from consideration. Although we included 23 studies to pool the results, the substantial heterogeneity cannot be overlooked. Despite performing a series of sensitivity analyses and meta-regressions, we were unable to accurately identify all the possible reasons for the substantial heterogeneity observed in our results. Additionally, due to the substantial heterogeneity, which could strongly influence the true effect, we chose to perform an exploratory subgroup analysis. These limitations should be considered when interpreting our meta-analysis’ findings.

### Future research

We obtained the predictive effect interval for future observational studies by reducing heterogeneity through sensitivity analysis and meta regression. However, due to the inherent limitations of observational studies, the association between IBD and CVD currently cannot support primary prevention strategies. Therefore, randomized clinical trials are needed. The future trials may focus on the community population and the accumulated dose of IBD-modify medications such as corticosteroids. These factors can help answer whether CVD risk comes from IBD itself or from systemic inflammatory reactions caused by IBD activity. Similarly, designing studies to explore the reasons for the lack of association between IBD and CVD mortality is equally important.

## Conclusions

The purpose of this meta-analysis is to conduct a meta-analysis on whether inflammatory bowel disease is positively correlated with an increased risk of cardiovascular disease, and to clarify whether these patients without other cardiovascular risks require primary prevention with medications. After evaluating 23 observational studies (comprising 20 different samples) with a total of 45,887,104 participants, we observed that inflammatory bowel disease may increase the risk of cardiovascular disease. However, due to the low certainty of the evidence, we are unable to provide any clinical recommendations. Therefore, based on current evidence, it is not recommended to use medication for primary prevention. High quality randomized controlled trials are needed to answer this question.

## Supporting information

S1 Fig Baujat plot. S1A, Total IBD; S1B, CD; S1C, UC; S1D, IBD-U.
(DOCX)

S2 Fig Influence plot. S2A, Total IBD; S2B, CD; S2C, UC; S2D, IBD-U.
(DOCX)

S3 Fig Leave-one-out analysis. S3A, Total IBD; S3B, CD; S3C, UC; S3D, IBD-U.
(DOCX)

S4 Fig Funnel plot. S4A, Total IBD; S4B, CD; S4C, UC; S4D, IBD-U.
(DOCX)

S5 Fig Contour-Enhanced Funnel Plot. S5A, Total IBD; S5B, CD; S5C, UC.
(DOCX)

S6 Fig P-curve. S6A, Total IBD; S6B, CD; S6C, UC; S6D, IBD-U.
(DOCX)

S1 Table Detailed characteristics of the included studies. (XLSX)

S2 Table NOS scores of included studies. (DOCX)

S3 Table GRADE: Certainty of the evidence. (DCOX)

S4 Table Egger’s test. S4-1, Total IBD-CVDs; S4-2, CD-CVDs; S4-3, UC-CVDs.
(DOCX)

S5 Table Subgroup analysis. (DOCX)

S1 File PRISMA2020 checklist. (DOCX)

S2 File Meta-regression analysis. (DOCX)
