# Supplementary figures and images for: Strategies for assessing and preventing cardiovascular disease risk in inflammatory bowel disease patients: A meta-analysis and meta-regression and bibliometric review
Source: PLoS One. 2025 Jul 28;20(7):e0327734. doi: 10.1371/journal.pone.0327734 (PMC12303265; doi:10.1371/journal.pone.0327734)

**Figure S1 Baujat plot**

**A．Total IBD**

**
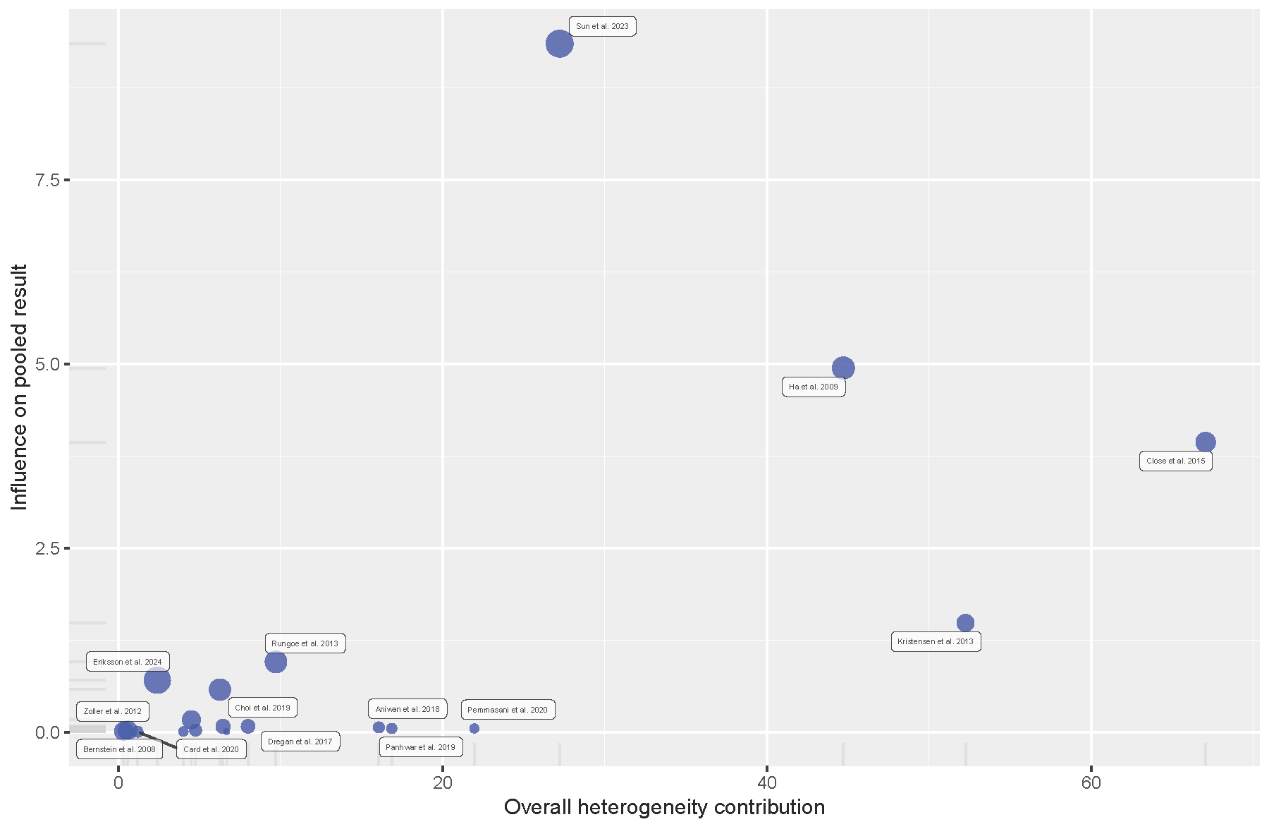
**

**B． CD**

**
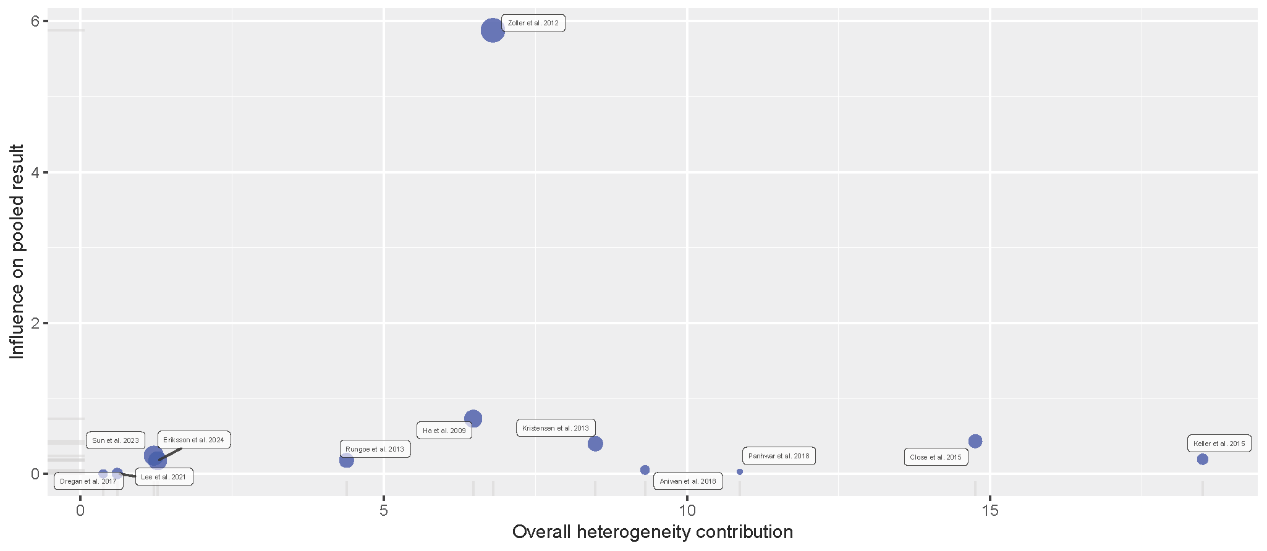
**

**C．UC**

**
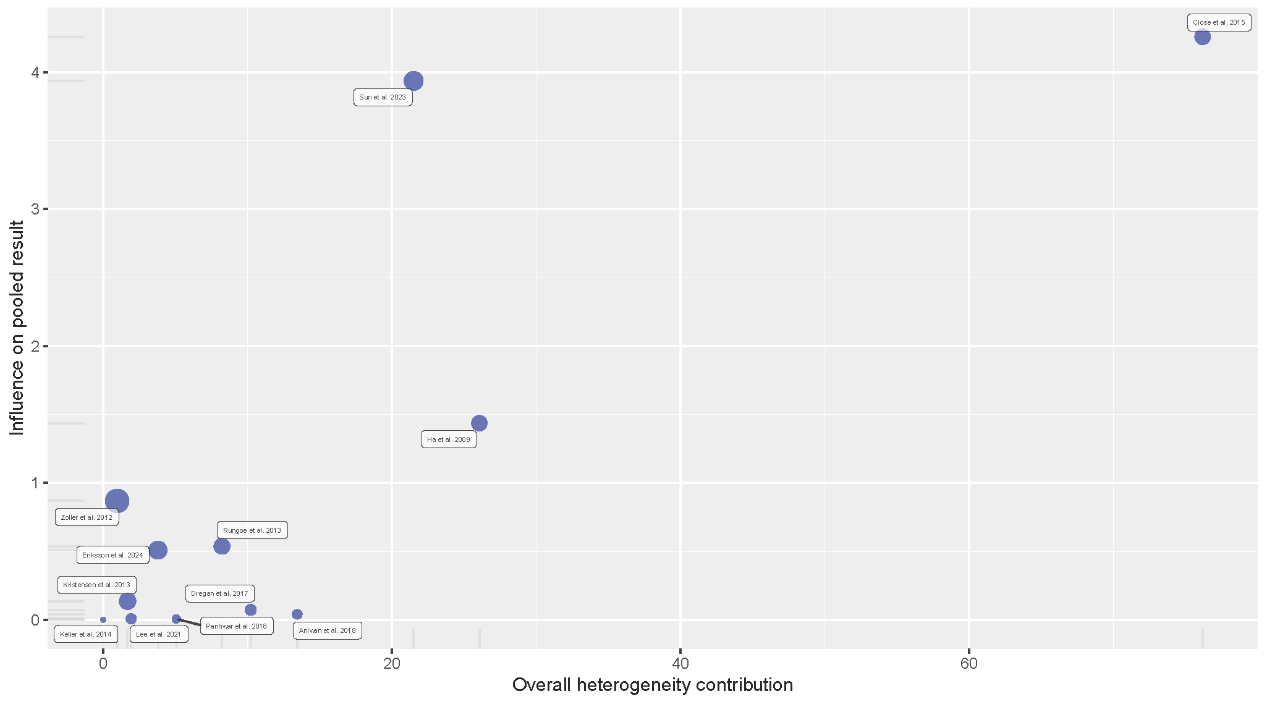
**

**D. IBD-U**

**
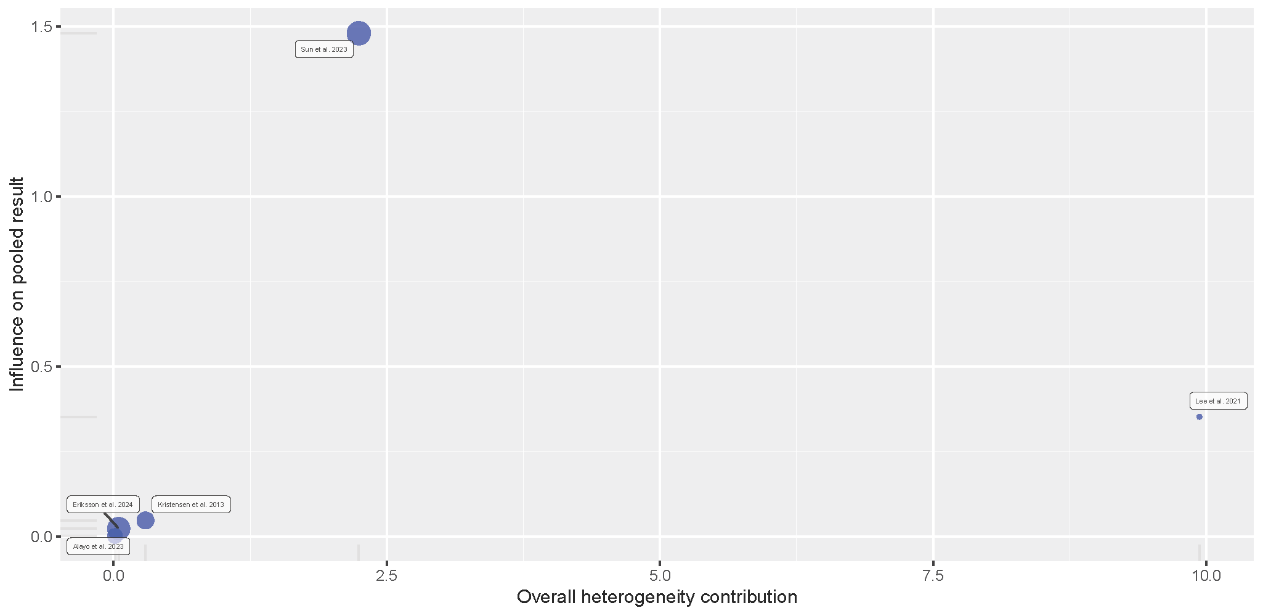
**

Supplement: S1 Fig — S1A, Total IBD; S1B, CD; S1C, UC; S1D, IBD-U. (DOCX) [file pone.0327734.s001.docx]

**Figure S2 Influence plot**

**
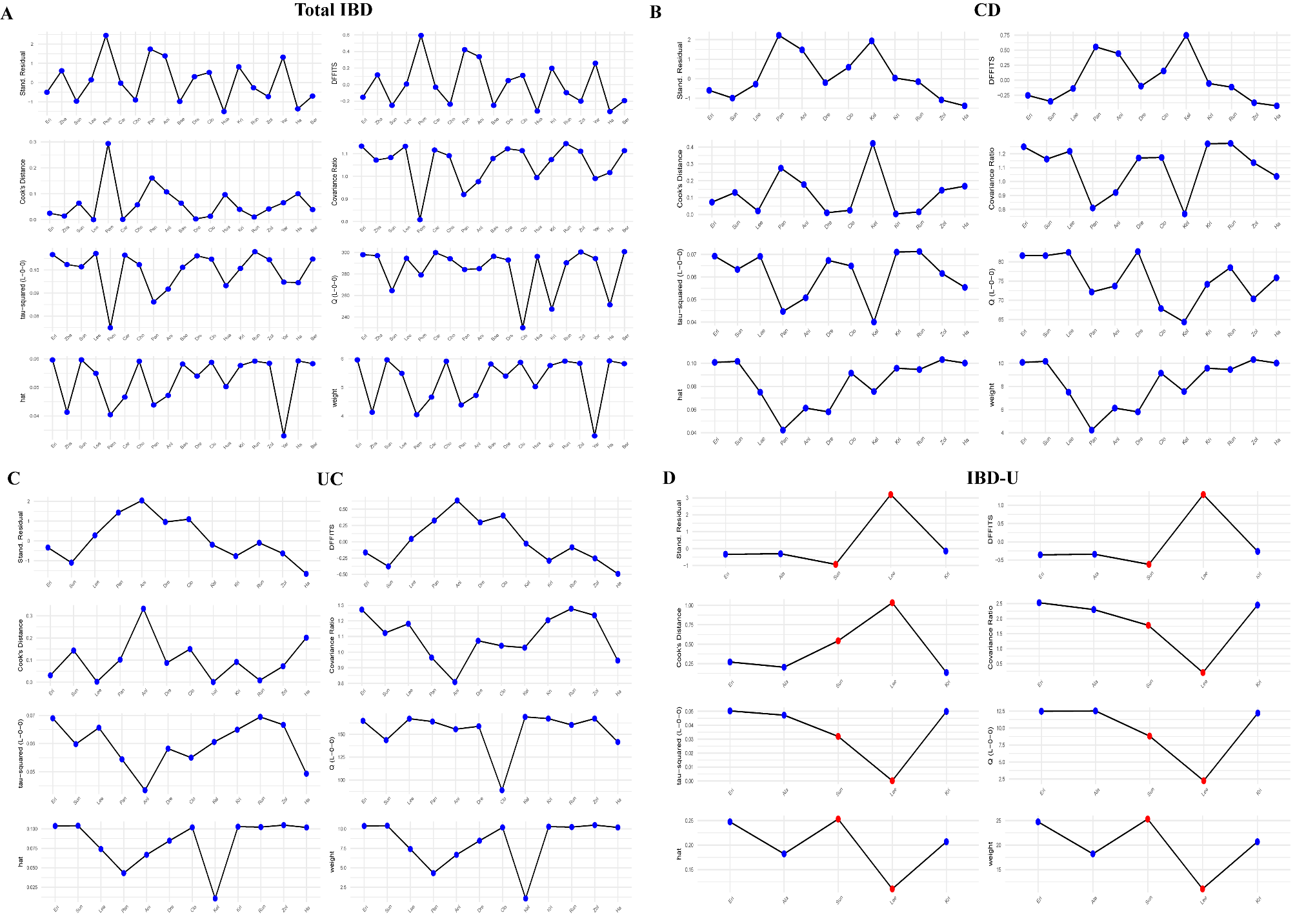
**

Supplement: S2 Fig — S2A, Total IBD; S2B, CD; S2C, UC; S2D, IBD-U. (DOCX) [file pone.0327734.s002.docx]

**Figure S3 Leave-one-out analysis**

**
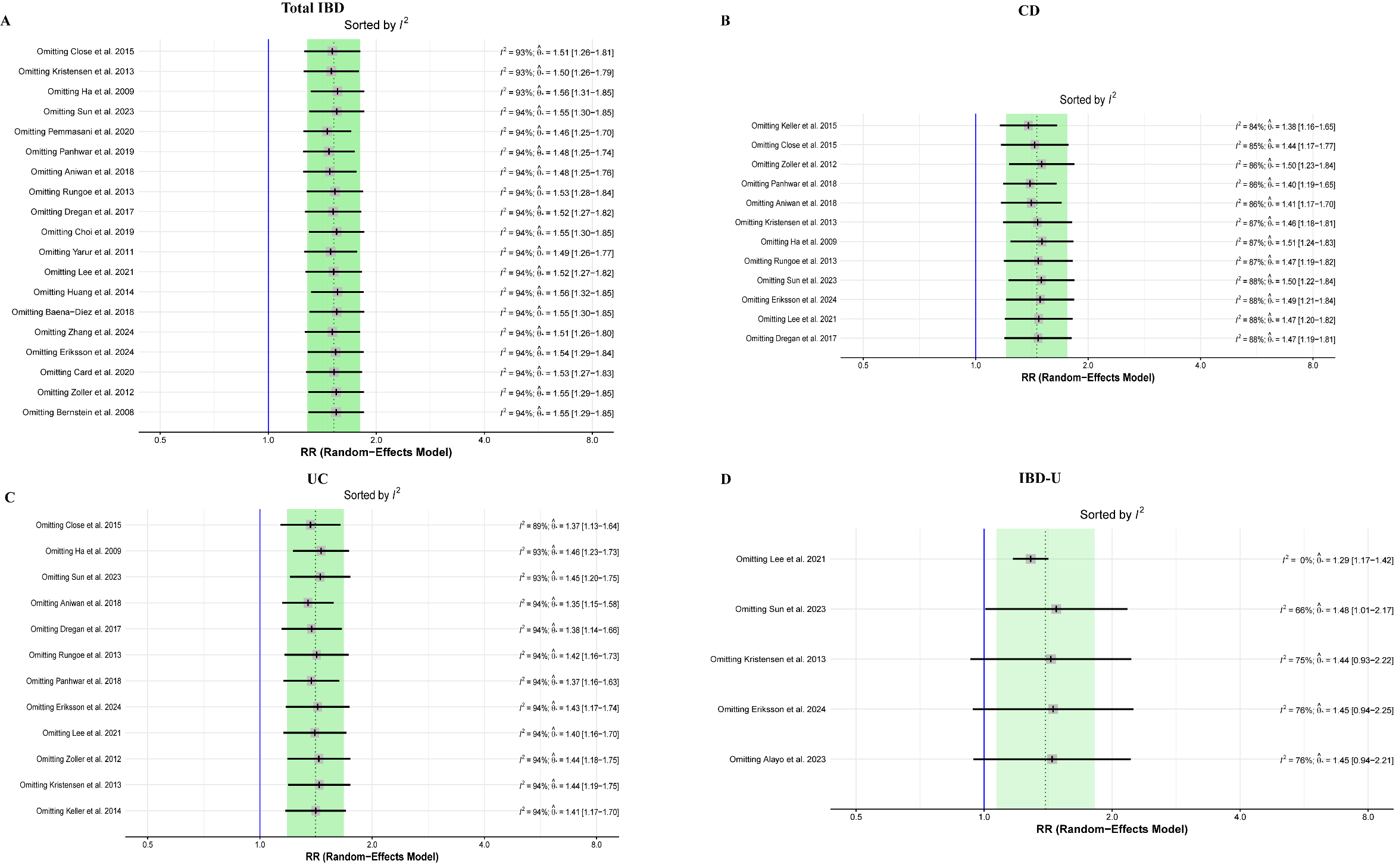
**

Supplement: S3 Fig — S3A, Total IBD; S3B, CD; S3C, UC; S3D, IBD-U. (DOCX) [file pone.0327734.s003.docx]

**S4 Figure Funnel plot**

**
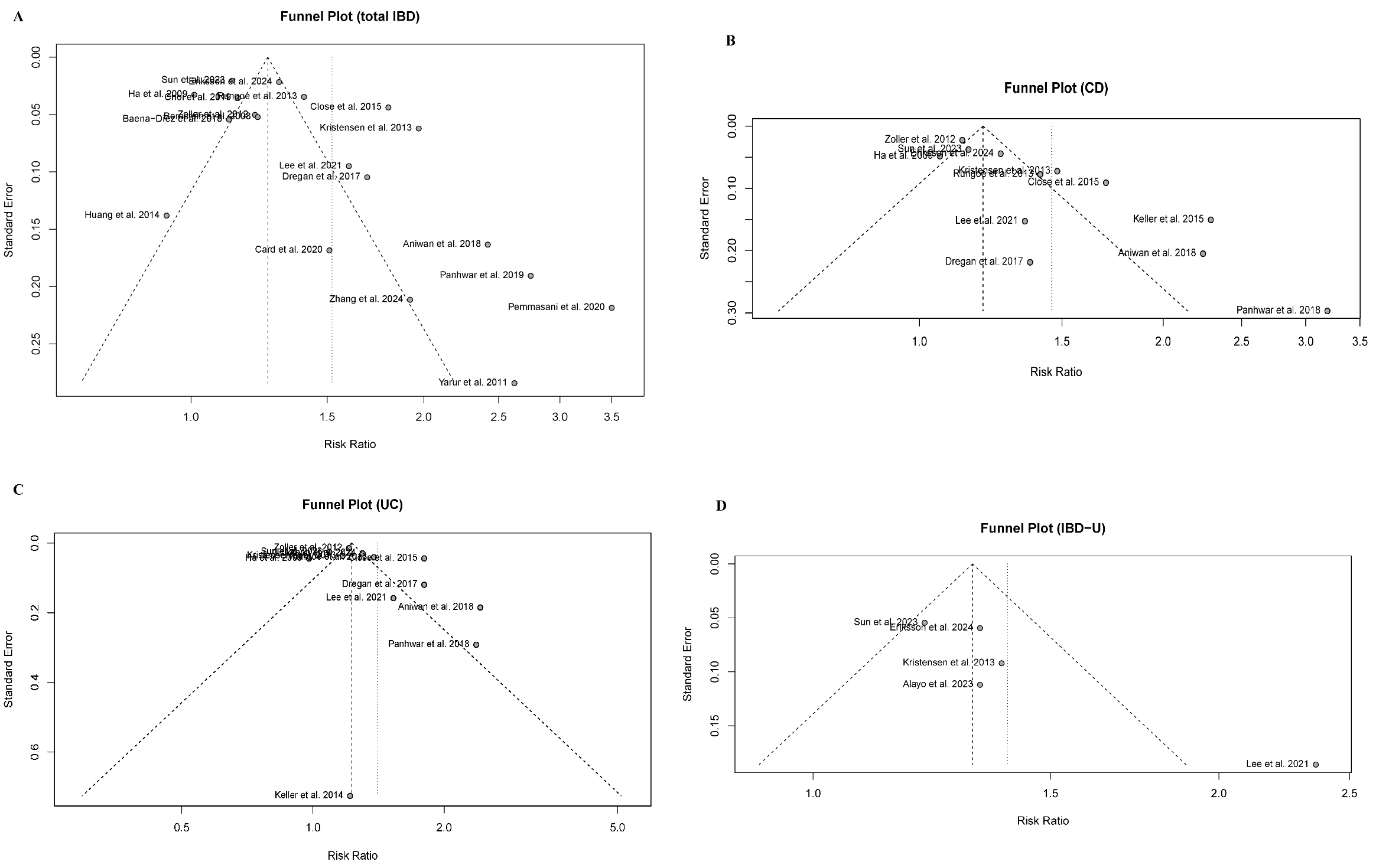
**

Supplement: S4 Fig — S4A, Total IBD; S4B, CD; S4C, UC; S4D, IBD-U. (DOCX) [file pone.0327734.s004.docx]

**S5 Figure Contour-Enhanced Funnel Plot**

**
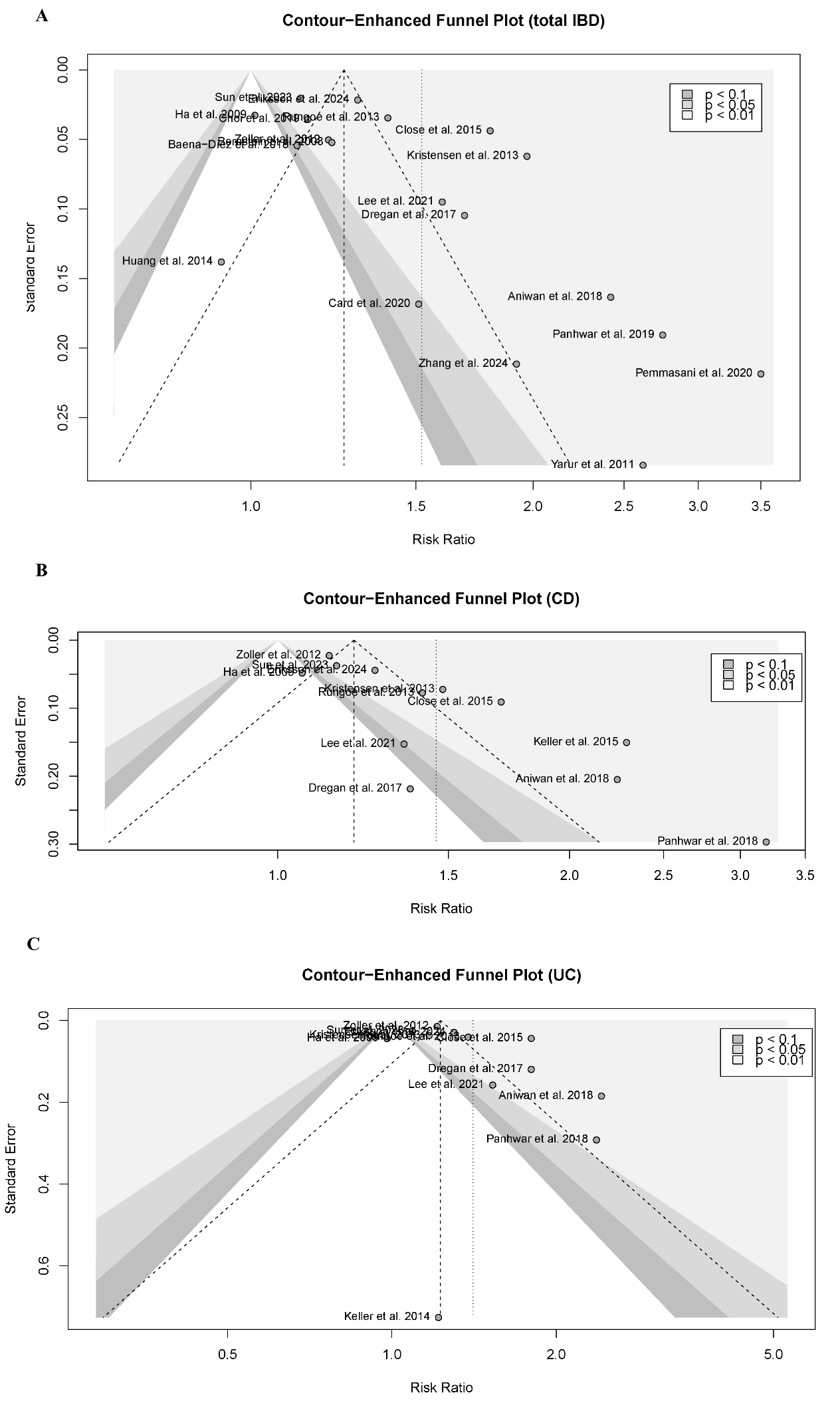
**

Supplement: S5 Fig — S5A, Total IBD; S5B, CD; S5C, UC. (DOCX) [file pone.0327734.s005.docx]

**S6 Figure *P*-curve**

**
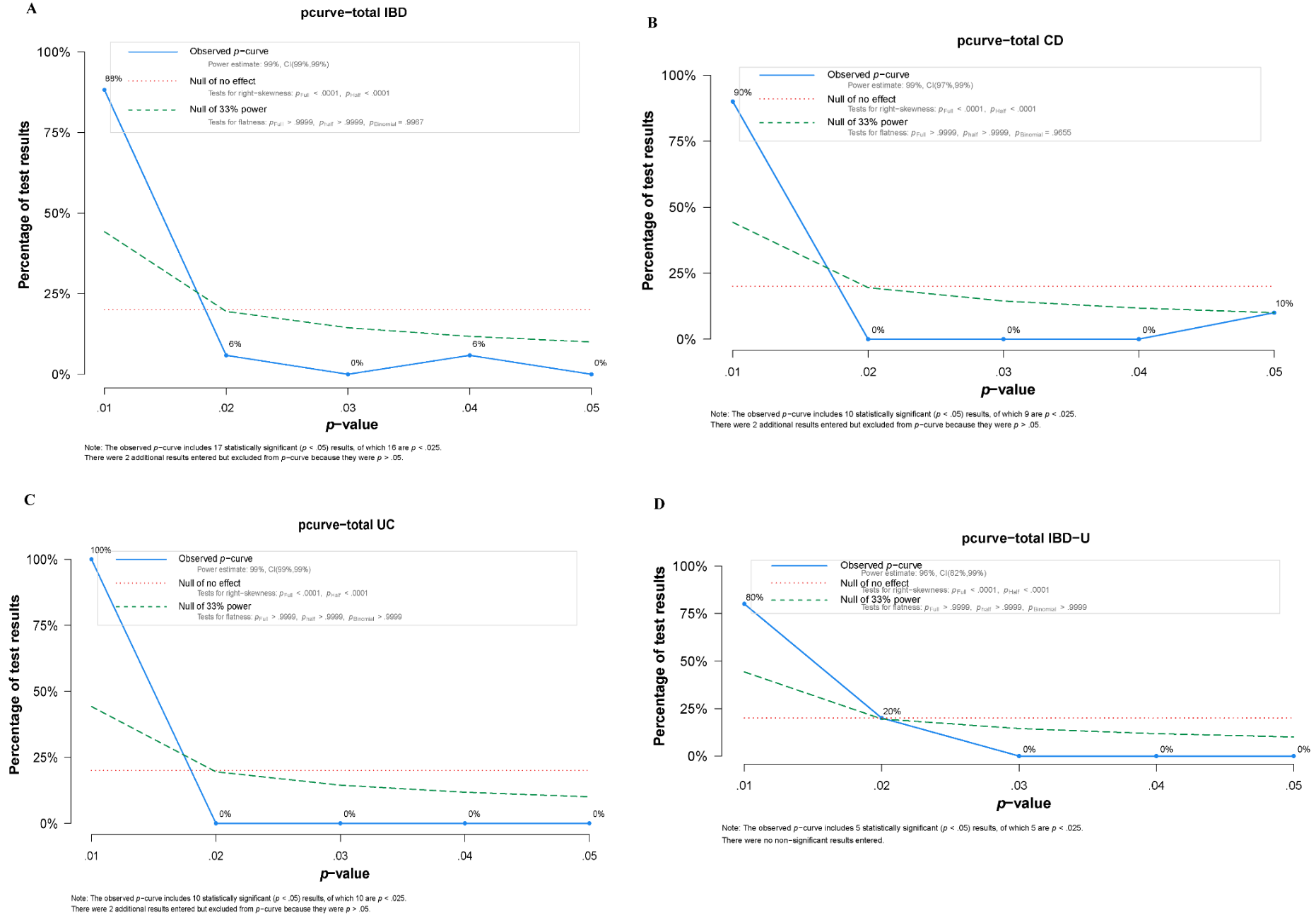
**

Supplement: S6 Fig — S6A, Total IBD; S6B, CD; S6C, UC; S6D, IBD-U. (DOCX) [file pone.0327734.s006.docx]
