# Supplementary material for: Strategies for assessing and preventing cardiovascular disease risk in inflammatory bowel disease patients: A meta-analysis and meta-regression and bibliometric review
Source: PLoS One. 2025 Jul 28;20(7):e0327734. doi: 10.1371/journal.pone.0327734 (PMC12303265; doi:10.1371/journal.pone.0327734)
Supplement: S2 Table — (DOCX) [file pone.0327734.s008.docx]

**Quality Assessment of Included Studies by Newcastle–Ottawa Scales**

| **Study** |  | **Selection** | | | | | | | | **Comparability** | | **Outcome** | | | | **Total**  **Score** |
| --- | --- | --- | --- | --- | --- | --- | --- | --- | --- | --- | --- | --- | --- | --- | --- | --- |
|  | **Year** | **Exposed**  **Cohort** | | **Non exposed**  **Cohort** | | **Ascertainment**  **of Exposure** | | **Outcome**  **of Interest** | |  |  | **Assessment**  **of Outcome** | | **Length of**  **Follow-up** | **Adequacy**  **of Follow-up** |  |
| Eriksson et al. | 2024 | ★ | ★ | |  | |  | | ★ | | ★ | | ★ | | ★ | 6 |
| Card et al. | 2020 | ★ | ★ | | ★ | | ★ | | ★★ | | ★ | | ★ | | ★ | 9 |
| Choi et al. | 2019 | ★ | ★ | | ★ | | ★ | | ★ | | ★ | |  | |  | 6 |
| Baena-Díez et al. | 2018 | ★ | ★ | | ★ | | ★ | | ★ | | ★ | |  | |  | 6 |
| Aniwan et al. | 2018 | ★ | ★ | | ★ | | ★ | | ★ | | ★ | | ★ | |  | 7 |
| Dregan et al. | 2017 | ★ | ★ | | ★ | | ★ | | ★ | | ★ | | ★ | | ★ | 8 |
| Close et al. | 2015 | ★ | ★ | | ★ | | ★ | | ★ | | ★ | | ★ | | ★ | 8 |
| Dregan et al. | 2017 | ★ | ★ | | ★ | | ★ | | ★★ | | ★ | | ★ | | ★ | 9 |
| Ha et al. | 2009 | ★ | ★ | | ★ | | ★ | | ★ | | ★ | | ★ | |  | 7 |
| Bernstein et al. | 2008 | ★ | ★ | | ★ | | ★ | | ★ | | ★ | | ★ | |  | 7 |
| Eriksson et al. | 2024 | ★ | ★ | | ★ | | ★ | | ★ | | ★ | | ★ | | ★ | 8 |
| Huang, Wei-Shih et al. | 2014 | ★ | ★ | |  | |  | | ★ | | ★ | | ★ | | ★ | 6 |
| Keller et al. | 2014 | ★ | ★ | |  | |  | | ★★ | | ★ | | ★ | | ★ | 7 |
| Keller et al. | 2015 | ★ | ★ | |  | |  | | ★ | | ★ | | ★ | | ★ | 6 |
| Kristensen et al. 2013 | 2013 | ★ | ★ | | ★ | | ★ | | ★ | | ★ | | ★ | | ★ | 8 |
| Alayo et al. | 2023 | ★ | ★ | | ★ | |  | | ★ | | ★ | | ★ | | ★ | 7 |
| Lee et al. | 2021 | ★ | ★ | | ★ | | ★ | | ★★ | | ★ | | ★ | | ★ | 9 |
| Panhwar et al. | 2018 | ★ | ★ | | ★ | | ★ | | ★ | | ★ | |  | |  | 6 |
| Pemmasani et al. | 2020 | ★ | ★ | | ★ | | ★ | | ★ | | ★ | |  | |  | 6 |
| Sun et al. | 2023 | ★ | ★ | | ★ | | ★ | | ★ | | ★ | |  | |  | 6 |
| Yarur et al. | 2011 | ★ | ★ | | ★ | | ★ | | ★ | | ★ | | ★ | | ★ | 8 |
| Zoller et al. | 2012 | ★ | ★ | | ★ | | ★ | | ★ | | ★ | | ★ | | ★ | 8 |
| Rungoe et al. | 2013 | ★ | ★ | | ★ | | ★ | | ★★ | | ★ | | ★ | | ★ | 9 |
| Zhang et al | 2024 | ★ | ★ | | ★ | | ★ | | ★ | | ★ | | ★ | | ★ | 8 |
