# Supplementary material for: Strategies for assessing and preventing cardiovascular disease risk in inflammatory bowel disease patients: A meta-analysis and meta-regression and bibliometric review
Source: PLoS One. 2025 Jul 28;20(7):e0327734. doi: 10.1371/journal.pone.0327734 (PMC12303265; doi:10.1371/journal.pone.0327734)
Supplement: S3 Table — (DCOX) [file pone.0327734.s009.docx]

| **Certainty assessment** | | | | | | | **№ of patients** | | **Effect** | | **Certainty** | **Importance** |
| --- | --- | --- | --- | --- | --- | --- | --- | --- | --- | --- | --- | --- |
| **№ of studies** | **Study design** | **Risk of bias** | **Inconsistency** | **Indirectness** | **Imprecision** | **Other considerations** | **IBD** | **CVD** | **Relative (95% CI)** | **Absolute (95% CI)** |  |  |
| **Cardiovascular Disease (assessed with: total IBD)** | | | | | | | | | | | | |
| 19 | non-randomised studies | not serious^a^ | not serious | serious^b^ | serious^c^ | publication bias strongly suspected all plausible residual confounding would suggest spurious effect, while no effect was observed^d^ | 0/0 | 0.0% | **RR 1.52** (1.28 to 1.80) | **0 fewer per 1,000** (from 0 fewer to 0 fewer) | ⨁◯◯◯ Very low^a,b,c,d^ |  |
| **Cardiovascular Disease (assessed with: CD)** | | | | | | | | | | | | |
| 12 | non-randomised studies | not serious | not serious | serious^e^ | serious^c^ | publication bias strongly suspected all plausible residual confounding would suggest spurious effect, while no effect was observed^d^ | 0/0 | 0/0 | **RR 1.46** (1.21 to 1.76) | **1 fewer per 1,000** (from 2 fewer to 1 fewer) | ⨁◯◯◯ Very low^c,d,e^ |  |
| **Cardiovascular Disease (assessed with: UC)** | | | | | | | | | | | | |
| 12 | non-randomised studies | not serious | not serious | serious^f^ | serious^c^ | all plausible residual confounding would reduce the demonstrated effect | 0/0 | 0/0 | **RR 1.41** (1.18 to 1.68) | **1 fewer per 1,000** (from 2 fewer to 1 fewer) | ⨁◯◯◯ Very low^c,f^ |  |
| **Cardiovascular Disease (assessed with: IBD-U)** | | | | | | | | | | | | |
| 5 | non-randomised studies | not serious | not serious | serious^g^ | serious^c^ | all plausible residual confounding would reduce the demonstrated effect |  |  | **RR 1.29** (1.17 to 1.42) | **1 fewer per 1,000** (from 1 fewer to 1 fewer) | ⨁◯◯◯ Very low^c,g^ |  |
| **Acute myocardial infarction (assessed with: total IBD)** | | | | | | | | | | | | |
| 9 | non-randomised studies | not serious | serious^h^ | serious^i^ | not serious^c^ | all plausible residual confounding would suggest spurious effect, while no effect was observed |  |  | **RR 1.81** (1.34 to 2.43) | **2 fewer per 1,000** (from 2 fewer to 1 fewer) | ⨁◯◯◯ Very low^h,i^ |  |
| **Acute myocardial infarction (assessed with: CD)** | | | | | | | | | | | | |
| 6 | non-randomised studies | not serious | not serious | serious^j^ | serious^c^ | all plausible residual confounding would reduce the demonstrated effect |  |  | **RR 1.53** (1.14 to 2.05) | **2 fewer per 1,000** (from 2 fewer to 1 fewer) | ⨁◯◯◯ Very low^c,j^ |  |
| **Acute myocardial infarction (assessed with: UC)** | | | | | | | | | | | | |
| 6 | non-randomised studies | not serious | serious^h^ | serious^k^ | serious^c^ | all plausible residual confounding would reduce the demonstrated effect |  |  | **RR 1.37** (1.02 to 1.83) | **1 fewer per 1,000** (from 2 fewer to 1 fewer) | ⨁◯◯◯ Very low^h,k,l^ |  |
| **Acute myocardial infarction (assessed with: IBD-U)** | | | | | | | | | | | | |
| 2 | non-randomised studies | not serious | serious^m^ | serious^n^ | serious^c^ | all plausible residual confounding would reduce the demonstrated effect |  |  | **RR 1.24** (1.11 to 1.39) | **1 fewer per 1,000** (from 1 fewer to 1 fewer) | ⨁◯◯◯ Very low^c,m,n^ |  |
| **Stroke (assessed with: IBD)** | | | | | | | | | | | | |
| 10 | non-randomised studies | not serious | serious^h^ | serious^o^ | serious^m^ | all plausible residual confounding would suggest spurious effect, while no effect was observed |  |  | **RR 1.17** (1.06 to 1.30) | **1 fewer per 1,000** (from 1 fewer to 1 fewer) | ⨁◯◯◯ Very low^h,m,o^ |  |
| **Stroke (assessed with: CD)** | | | | | | | | | | | | |
| 5 | non-randomised studies | not serious | not serious | serious^p^ | serious^c^ | all plausible residual confounding would suggest spurious effect, while no effect was observed |  |  | **RR 1.39** (0.99 to 1.93) | **1 fewer per 1,000** (from 2 fewer to 1 fewer) | ⨁◯◯◯ Very low^c,p^ |  |
| **Stroke (assessed with: UC)** | | | | | | | | | | | | |
| 5 | non-randomised studies | not serious | serious^h^ | serious^q^ | serious^c^ | all plausible residual confounding would suggest spurious effect, while no effect was observed |  |  | **RR 1.12** (1.03 to 1.22) | **1 fewer per 1,000** (from 1 fewer to 1 fewer) | ⨁◯◯◯ Very low^c,h,q^ |  |
| **Stroke (assessed with: IBD-U)** | | | | | | | | | | | | |
| 1 | non-randomised studies | not serious | not serious | serious^r^ | serious^c^ | all plausible residual confounding would reduce the demonstrated effect |  |  | **RR 1.41** (1.06 to 1.88) | **1 fewer per 1,000** (from 2 fewer to 1 fewer) | ⨁◯◯◯ Very low^c,r^ |  |

**CI:** confidence interval; **RR:** risk ratio

#### Explanations

a. the publication bias cannot be ignored

b. The cohort study cannot tell the causal relationship but give the possible association between IBD and CVD risk

c. then population was not clearly defined.

d. the publication bias existed

e. The cohort study cannot tell the causal relationship but give the possible association between CD and CVD risk

f. The cohort study cannot tell the causal relationship but give the possible association between UC and CVD risk

g. The cohort study cannot tell the causal relationship but give the possible association between IBD-U and CVD risk

h. the included studies showed the different effect

i. The cohort study cannot tell the causal relationship but give the possible association between IBD and AMI risk

j. The cohort study cannot tell the causal relationship but give the possible association between CD and AMI risk

k. The cohort study cannot tell the causal relationship but give the possible association between UC and AMI risk

l. The cohort study cannot tell the causal relationship but give the possible association between UC and AMI risk

m. the results were not consistent

n. The cohort study cannot tell the causal relationship but give the possible association between IBD-U and AMI risk

o. The cohort study cannot tell the causal relationship but give the possible association between IBD and stroke risk

p. The cohort study cannot tell the causal relationship but give the possible association between CD and stroke risk

q. The cohort study cannot tell the causal relationship but give the possible association between UC and stroke risk

r. The cohort study cannot tell the causal relationship but give the possible association between IBD-U and stroke risk
