# Supplementary material for: Strategies for assessing and preventing cardiovascular disease risk in inflammatory bowel disease patients: A meta-analysis and meta-regression and bibliometric review
Source: PLoS One. 2025 Jul 28;20(7):e0327734. doi: 10.1371/journal.pone.0327734 (PMC12303265; doi:10.1371/journal.pone.0327734)
Supplement: S4 Table — S4-1, Total IBD-CVDs; S4-2, CD-CVDs; S4-3, UC-CVDs. (DOCX) [file pone.0327734.s010.docx]

**S4-1 Table (Total IBD-CVDs)**

|  | Intercept | CI | t | P |
| --- | --- | --- | --- | --- |
| Egger's test | 3.448 | 0.78 - 6.12 | 2.531 | 0.02 |

**S4-2 Table (CD-CVDs)**

|  | Intercept | CI | t | P |
| --- | --- | --- | --- | --- |
| Egger's test | 3.491 | 1.95 – 5.03 | 4.432 | 0.001 |

**S4-3 Table (UC-CVDs)**

|  | Intercept | CI | t | P |
| --- | --- | --- | --- | --- |
| Egger's test | 2.42 | -0.87 – 5.71 | 1.44 | 0.18 |
