# Supplementary material for: Strategies for assessing and preventing cardiovascular disease risk in inflammatory bowel disease patients: A meta-analysis and meta-regression and bibliometric review
Source: PLoS One. 2025 Jul 28;20(7):e0327734. doi: 10.1371/journal.pone.0327734 (PMC12303265; doi:10.1371/journal.pone.0327734)
Supplement: S5 Table — (DOCX) [file pone.0327734.s011.docx]

**Table S5. Exploratory subgroup analysis**

| **Subgroup** | **RR (95%CI)** | **I^2^** | **P for interaction** |
| --- | --- | --- | --- |
| **Publication year** |  |  |  |
| 2008−2015 | 1.35 (1.12-1.64) | 95.7% | 0.29 |
| 2016−2024 | 1.57 (1.28-1.94) | 91.2% |  |
| **Follow-up** |  |  |  |
| <10 years | 1.65 (1.18-2.31) | 95.1% | 0.42 |
| ≥10 years | 1.44 (1.18-1.75) | 93.5% |  |
| **Continents** |  |  |  |
| Europe | 1.44 (1.24-1.68) | 94.7% | 0.0065 |
| North America | 1.91 (1.24-2.94) | 94.3% |  |
| Asia | 1.08 (0.31-3.72) | 54.9% |  |
| **Risk assessment indicators adjusted** |  |  |  |
| NO | 3.14 (2.24-4.41) | 0 | <0.001 |
| YES | 1.43 (1.23-1.66) | 94.1% |  |
| **Adequate adjustment for confounding factors** |  |  |  |
| YES | 1.42 (1.18-1.72) | 95.1% | 0.31 |
| NO | 1.72 (1.15-2.59) | 91.2% |  |
