# Supplementary material for: Strategies for assessing and preventing cardiovascular disease risk in inflammatory bowel disease patients: A meta-analysis and meta-regression and bibliometric review
Source: PLoS One. 2025 Jul 28;20(7):e0327734. doi: 10.1371/journal.pone.0327734 (PMC12303265; doi:10.1371/journal.pone.0327734)
Supplement: S2 File — (DOCX) [file pone.0327734.s013.docx]

**Meta-regression**

| **Total IBD-CVDs** | | | | | |
| --- | --- | --- | --- | --- | --- |
| Moderators | tau^2 | tau | I^2  residual heterogeneity / unaccounted variability | H^2  unaccounted variability / sampling variability | R^2  amount of heterogeneity accounted for |
| NOS | 0.013 | 0.114 | 26.99% | 1.37 | 76.73% |
| Age, NOS, Sample size, Publication year | 0 | 0.0014 | 0.01% | 1 | 100% |

| **Total CD-CVDs** | | | | | |
| --- | --- | --- | --- | --- | --- |
| Moderators | tau^2 | tau | I^2  residual heterogeneity / unaccounted variability | H^2  unaccounted variability / sampling variability | R^2  amount of heterogeneity accounted for |
| Sample size | 0 | 0 | 0 | 1 | 100% |
| Age, NOS, Sample size, Publication year | 0 | 0 | 0 | 1 | 100% |

| **Total UC-CVDs** | | | | | |
| --- | --- | --- | --- | --- | --- |
| Moderators | tau^2 | tau | I^2  residual heterogeneity / unaccounted variability | H^2  unaccounted variability / sampling variability | R^2  amount of heterogeneity accounted for |
| NOS | 0.02 | 0.12 | 38.83% | 1.63 | 44.71% |
| Age, NOS, Sample size, Publication year | 0 | 0 | 0 | 1 | 100% |

| Rücker’s Limit Meta-Analysis Method | | | | |
| --- | --- | --- | --- | --- |
| Random effects model | RR | 95%CI | z | *P* val |
| Adjusted estimate | 1.36 | 1.15-1.61 | 3.66 | 0.0003 |
| Unadjusted estimate | 1.41 | 1.18-1.68 | 4.29 | 0.0013 |

**IBDU-CVDs**

| Analysis | *RR* | 95%CI | *P* | 95%PI | *I^2^* | 95%CI |
| --- | --- | --- | --- | --- | --- | --- |
| Main Analysis | 1.39 | 1.07-1.82 | 0.0256 | 0.88-2.22 | 68.1 | 17.6-87.6 |
| Infl. Cases Removed-outliers^1^ | 1.29 | 1.17-1.42 | 0.0036 | 1.14-1.45 | 0 | 0-84.7 |

1. Results with outliers removed: "Lee et al. 2021"
